# Whole-Genome Sequence of Multidrug-Resistant Escherichia coli Strains Isolated from Mice with Mastitis

**DOI:** 10.1128/mra.00320-23

**Published:** 2023-06-14

**Authors:** M. Nazmul Hoque, Shobnom Jerin, Golam Mahbub Faisal, Ziban Chandra Das, Tofazzal Islam, Abu Nasar M. Aminoor Rahman

**Affiliations:** a Molecular Biology and Bioinformatics Laboratory, Department of Gynaecology, Obstetrics, and Reproductive Health, Bangabandhu Sheikh Mujibur Rahman Agricultural University, Gazipur, Bangladesh; b Institute of Biotechnology and Genetic Engineering, Bangabandhu Sheikh Mujibur Rahman Agricultural University, Gazipur, Bangladesh; Indiana University, Bloomington

## Abstract

We sequenced the genomes of Escherichia coli isolates G2M6U and G6M1F, two multidrug-resistant strains that were isolated from mammary tissue and fecal samples, respectively, from mice with induced mastitis. The complete genomes of G2M6U and G6M1F consist of chromosomes of 4.4 Mbp and 4.6 Mbp, respectively.

## ANNOUNCEMENT

Escherichia coli is a rod-shaped, flagellated, facultative aerobic, nonsporulating, Gram-negative bacterium of the family *Enterobacteriaceae*. It is considered one of the most important causes of clinical and subclinical mastitis in dairy cows (1, 2). Clinical signs of E. coli mastitis range from severe peracute disease to subclinical and chronic infections, depending on the virulence of the invading strain(s) and the nutrition status and immune responses of affected cows (3–5). According to recent research, transferring microbiota from the feces and milk of cows with mastitis to pregnant mice resulted in the induction of mastitis, accompanied by dysbiosis of their microbiomes (6, 7). The emergence of antimicrobial-resistant E. coli strains isolated from dairy cow mastitis is increasing at an alarming pace, causing worldwide public health concerns (4). There is strong scientific evidence that connects the evolution of resistance in E. coli strains isolated from the guts of different host animals (e.g., mice, wild animals, and livestock) and humans (8–10).

The Animal Research Ethics Committee (AREC) of the Bangabandhu Sheikh Mujibur Rahman Agricultural University (Gazipur, Bangladesh) reviewed and approved the experimental procedures of this study (reference number FVMAS/AREC/2023/6679 [16 January 2023]). Mammary tissues and fecal samples from mice in which mastitis was induced (at our laboratory animal research facility [24.09°N, 90.41°E]) were plated on nutrient agar for overnight incubation at 37°C. Pure colonies were isolated and subsequently streaked on eosin methylene blue (EMB) agar (Oxoid; Thermo Fisher Scientific, UK) plates and incubated at 37°C for 24 h (4). Phenotypic identification of the isolates was performed based on the colony morphology and Gram staining results (Gram negative, with formation of a green metallic sheen on EMB agar) and the results of biochemical tests such as the catalase, indole, methyl red, Voges-Proskauer (VP), oxidase, urease, and triple sugar iron tests (4). The G2M6U and G6M1F isolates were confirmed as E. coli by 16S rRNA gene sequencing (4) and were found to be multidrug resistant (MDR) (resistant to >6 antibiotics) in disk diffusion tests performed according to CLSI guidelines (11). The MDR isolates of E. coli were incubated in nutrient broth (Biolife, Italy) overnight at 37°C, and the harvested cultures were used for DNA extraction with a QIAamp DNA minikit (Qiagen, Hilden, Germany). The DNA purity and concentration were checked using a NanoDrop 2000 UV-visible spectrophotometer (Thermo Fisher Scientific, Waltham, MA, USA). The Nextera DNA Flex library preparation kit (Illumina, San Diego, CA, USA) was used to generate libraries from 1 ng DNA, and whole-genome sequencing of the prepared libraries was performed using a MiSeq sequencer (Illumina) with a 2 × 250-bp protocol. The paired-end raw reads (G2M6U, 74,951,320 bp; G6M1F, 84,361,784 bp) were trimmed using Trimmomatic v0.39 (with parameters leading:20, slidingwindow:4:20:20, trailing:20, and minlen=36) to remove Illumina adapters, known Illumina artifacts, and phiX reads (12) and quality checked using FastQC v0.11.9 (13). Reads with Phred scores of >20 were assembled using SPAdes v3.15.5 (14), and the quality of the assembled genomes was checked using QUAST v5.0.2 (15). The NCBI Prokaryotic Genome Annotation Pipeline (PGAP) v6.4 (16) and BacWGSTdb v2.0 (17) were used for genome annotation and sequence typing, respectively. The ResFinder v4.0 (18), PlasmidFinder (19), Virulence Factor Database (VFDB) (20), and RAST FIGfams v70 (21) databases were used to predict antimicrobial resistance genes (ARGs), plasmids, virulence factors, and metabolic functions, respectively, in the assembled genomes. Default parameters were used for all software unless otherwise stated.

The draft assembly sizes of G2M6U and G6M1F were 4.4 Mbp and 4.6 Mbp, respectively. Further sequencing and assembly statistics are given in Table 1. These two isolates were typed as E. coli sequence type 155 (ST155) and ST58 according to seven-gene multilocus sequence typing (MLST) (*adk*, *fumC*, *gyrB*, *icd*, *mdh*, *purA*, and *recA*) with a core genome MLST (cgMLST) scheme. The RAST FIGfams v70 annotations revealed that the G2M6U genome contained 367 metabolic features in SEED subsystems, with 32% coverage, 4,343 protein-coding sequences (CDSs), and 68 RNA genes; whereas 378 SEED subsystem features, with 32% coverage, 4,607 CDSs, and 76 RNA genes, were predicted in the G6M1F genome. Seventy-eight ARGs (G2M6U, 45 ARGs; G6M1F, 41 ARGs), conferring resistance to multiple antibiotics and metals, and 276 virulence factor-related genes (G2M6U, 160 virulence factor-related genes; G6M1F, 178 virulence factor-related genes) were detected in the draft genomes. One plasmid replicon like IncY (4,012 bp) was identified in the G6M1F genome (with 95% identity and 60% coverage), while the G2M6U genome harbored none.

**TABLE 1:** Genomic features of Escherichia coli strains G2M6U and G6M1F, isolated from mice with mastitis

Parameter	Finding for strain:	
	G2M6U	G6M1F
Genome coverage (×)	60	65.5
Genome size (bp)	4,441,064	4,667,456
No. of contigs	41	57
*N*_50_ (bp)	140,681	184,919
GC content (%)	50.8	50.9
Genome accession no.	JARLTH000000000	JARLTG000000000
SRA accession no.	SRR23912641	SRR23912640

### Data availability.

Sequencing data were deposited in GenBank and the NCBI Sequence Read Archive (SRA) under BioProject accession number PRJNA946194. Individual accession numbers are listed in Table 1.
